# Multifaceted mechanisms of plant metabolites in pulmonary arterial hypertension: a critical review beyond vasodilation

**DOI:** 10.3389/fphar.2026.1769990

**Published:** 2026-03-05

**Authors:** Junjun Li, Chenyan Hu, Jia-Hui Zhu, Ruo-Lan Li

**Affiliations:** 1 Department of Pharmacy, Sichuan Clinical Research Center for Cancer, Sichuan Cancer Hospital & Institute, Sichuan Cancer Center, University of Electronic Science and Technology of China, Chengdu, China; 2 Department of Laboratory Medicine, Medical Center Hospital of Qionglai City, Chengdu, Sichuan, China; 3 Department of Clinical Research, Sichuan Clinical Research Center for Cancer, Sichuan Cancer Hospital & Institute, Sichuan Cancer Center, University of Electronic Science and Technology of China, Chengdu, China

**Keywords:** multi-target, pathogenesis, plant metabolites, pulmonary arterial hypertension, vascular remodeling

## Abstract

Pulmonary arterial hypertension (PAH) is a progressive vascular disease characterized by remodeling, inflammation, and metabolic dysregulation. Current pharmacotherapies primarily target vasodilation but fail to reverse structural remodeling or arrest disease progression. Plant metabolites have been proposed as potential therapeutic leads due to their structural diversity and reported multi-target actions; however, their safety and efficacy profiles in PAH remain incompletely validated. Beyond vasodilation, plant metabolites have been reported to modulate vascular remodeling, inflammation, oxidative stress, cellular metabolism, and epigenetic regulation, predominantly in preclinical models. However, most supporting evidence remains preclinical, often derived from rodent models and high-concentration *in vitro* assays, with limited validation of direct target engagement and clinical translatability. This review critically evaluates the multifaceted mechanisms of plant metabolites in PAH beyond vasodilation, with an explicit focus on the quality of evidence, the relevance of preclinical models, and the significant confounding issue of pan-assay interference compounds (PAINS). We highlight that while many metabolites show promising multi-target effects *in vitro* and in rodent models, the translational potential of most is severely limited by unvalidated target engagement, poor pharmacokinetics, and a lack of rigorous clinical data.

## 1 Introduction

Pulmonary arterial hypertension (PAH) is a severe and progressive cardiopulmonary disease primarily characterized by sustained elevation of pulmonary vascular resistance (PVR). The core pathophysiological feature of PAH is pulmonary vascular remodeling, characterized by dysregulated proliferation, migration, and apoptosis resistance of vascular cells, accompanied by endothelial dysfunction, inflammation, extracellular matrix accumulation (ECM), and *in situ* thrombosis (Evans et al., 2021; Ghofrani et al., 2024; Thenappan et al., 2018). These changes are largely refractory to current therapies and ultimately culminate in right ventricular failure (Hassoun, 2021; Lewis et al., 2024; Schermuly et al., 2011). Although the incidence of PAH is relatively low, the disease is associated with disproportionately high morbidity and mortality (Ruopp and Cockrill, 2022).

Current therapeutic strategies for PAH are largely based on pharmacological vasodilation to reduce pulmonary arterial pressure and improve exercise capacity (Ghofrani et al., 2024; Zolty, 2021). These therapies primarily target three vasoactive signaling pathways: endothelin-1 (ET-1), nitric oxide (NO), and prostacyclin (Barnes et al., 2019; Benza et al., 2024; Chin et al., 2024; Lyle et al., 2017; Moutchia et al., 2024). Accordingly, approved drugs are categorized into endothelin receptor antagonists, phosphodiesterase-5 (PDE5) inhibitors, soluble guanylate cyclase (sGC) stimulators, and prostacyclin analogues.

Although these agents effectively alleviate vasoconstriction and improve hemodynamics, their mechanisms are largely single-target and symptom-oriented (Chin et al., 2024; Grünig et al., 2024; Moutchia et al., 2024). Crucially, most existing therapies fail to reverse established pulmonary vascular remodeling or adequately address other key pathological drivers of PAH, such as persistent inflammation, oxidative stress, metabolic dysregulation, and endothelial senescence (Atanasov et al., 2021; Martin de Miguel et al., 2023; Zolty, 2023). This fundamental limitation explains why disease progression often continues despite optimized vasodilatory treatment.

Recent advances, exemplified by sotatercept targeting the bone morphogenetic protein (BMP) activin signaling axis, indicate a paradigm shift toward disease-modifying therapies (Hoeper et al., 2023; Rubin and Naeije, 2023). Clinical trials have demonstrated sustained improvements in exercise capacity and biomarkers (Humbert et al., 2021; Preston et al., 2025). Nevertheless, PAH remains a refractory disease, and even emerging agents do not fully prevent advanced vascular remodeling or eliminate systemic side effects, highlighting the continued unmet clinical need.

Plant metabolites, particularly bioactive compounds derived from medicinal plants and Traditional Chinese Medicine (TCM), have been extensively explored as sources of hypothesis-generating leads for PAH due to their chemical diversity and reported pleiotropic effects (Atanasov et al., 2021; Cano-Prieto et al., 2024; He et al., 2025; Xiang et al., 2018; Yu et al., 2022; Zhang et al., 2021; Zeng et al., 2023). Unlike conventional synthetic drugs designed to act on single molecular targets, plant metabolites frequently modulate multiple signaling pathways simultaneously, making them particularly suitable for multifactorial diseases such as PAH (Wang et al., 2021; Xie et al., 2024; Zhang et al., 2023).

With advancements in modern technology, an increasing number of plant metabolites have been identified as significant candidates for PAH treatment. This review critically summarizes the multifaceted mechanisms of plant metabolites in PAH beyond vasodilation, with a focus on evidence quality, taxonomic clarity, and PAINS-related considerations. It highlights that, similar to existing pharmacological therapies, plant metabolites induce vasodilation and reduce pulmonary arterial pressure by regulating the balance of vasoactive substances. In addition, they act on PASMC through ion channels to regulate vascular tone. More importantly, plant metabolites directly target vascular remodeling, the core pathological feature of PAH, thereby improving vascular structure. Moreover, plant metabolites exert protective effects by acting on multiple targets, including inflammatory infiltration, oxidative stress, and metabolic reprogramming (Xue Z. et al., 2021). Despite these theoretical advantages, evidence supporting the role of plant metabolites in PAH is heterogeneous and predominantly preclinical. Many studies rely on rodent models or high-dose *in vitro* systems, and some commonly studied polyphenols may exhibit PAINS-related assay interference. These limitations necessitate cautious interpretation of mechanistic claims and underscore the importance of rigorous appraisal of evidence.

For this review, we conducted a comprehensive search of scientific electronic databases, including PubMed, Web of Science, and ScienceDirect, using keywords such as “pulmonary arterial hypertension”, “pathogenesis”, “plant metabolites”, “vascular remodeling”, “multi-target mechanisms”, and specific plant names covering the period from 2010 to 2025. The literature collection primarily focused on the therapeutic effects of plant metabolites in PAH. Inclusion criteria focused on studies providing clear mechanistic insights, specific dosage information, and validated animal or cell models. Manuscripts limited to abstracts or drafts, studies lacking specific doses and utilizing unverified extracts were excluded. In this review, we systematically summarize recent advances in the application of plant metabolites for the treatment of PAHs. In addition, the methodological quality of included studies was critically assessed with attention to animal model relevance, experimental design (randomization and blinding), choice of endpoints, and translational exposure relevance. A paramount consideration in this review is the PAINS alert. Many plant metabolites, especially polyphenols commonly studied in PAH (e.g., quercetin, resveratrol, curcumin, luteolin), are known PAINS. They can produce false-positive results in high-throughput and pathway-focused assays through non-specific mechanisms, leading to erroneous conclusions about target specificity and therapeutic potential. Throughout this review, we explicitly flag such compounds, critically appraise studies involving them, and distinguish between hypothesis-generating observations and validated pharmacological effects.

We focus on their multi-target mechanisms, particularly their ability to inhibit vascular remodeling, suppress inflammation and oxidative stress, and restore metabolic homeostasis beyond regulating vascular tone. By integrating evidence from cellular, animal, and clinical studies, this review aims to provide a coherent framework for understanding the therapeutic potential, current limitations, and prospects of plant metabolites in PAH.

## 2 Pathogenesis beyond vasoconstriction of PAH

PAH is a complex disease that poses a serious threat to patients’ lives. Initially considered a condition solely related to vasoconstriction, recent research increasingly reveals that PAH is a multifactorial vascular disorder driven by excessive vascular cell proliferation, inflammation, and metabolic dysregulation (Lewis et al., 2024; Schermuly et al., 2011). Therefore, gaining a comprehensive understanding of its pathogenesis is crucial for developing safer and more effective treatment strategies.

### 2.1 The complex pathogenesis of PAH

The primary characteristic of PAH is persistent pathological changes in the pulmonary arteries, leading to pulmonary vascular remodeling. This transformation shifts the pulmonary arteries from a low-pressure, high-flow system into a high-pressure, high-resistance conduit (Hassoun, 2021; Schermuly et al., 2011). Remodeling affects all layers of the pulmonary artery wall, thereby increasing vascular resistance.

Pulmonary vascular remodeling in PAH evolves from early endothelial injury to sustained cellular proliferation, ECM, and ultimately the formation of complex plexiform lesions (Evans et al., 2021; Li X. et al., 2024; Shimoda, 2020; Tuder et al., 2024). These structural alterations are reinforced by persistent inflammation, oxidative stress, and metabolic reprogramming, creating a self-amplifying pathogenic network (Evans et al., 2021). EC is crucial for maintaining vascular homeostasis and is functionally impaired in the early stages of PAH. This damage not only impairs vasodilation but also renders ECs susceptible to pathological processes, including proliferation, inflammation, and thrombosis, driven by increased oxidative stress and metabolic dysfunction (Evans et al., 2021). Furthermore, EC senescence, characterized by irreversible cell-cycle arrest and the emergence of the senescence-associated secretory phenotype (SASP), contributes to endothelial dysfunction, inflammation, and abnormal vascular changes in PAH (Culley and Chan, 2022; Safaie Qamsari and Stewart, 2024).

The pathogenesis of PAH involves complex interactions between multiple molecular and cellular processes, including persistent inflammation, elevated oxidative stress, and metabolic dysregulation. Persistent inflammation is now recognized as a key driver of PAH development, as immune cell-derived factors promote vascular cell proliferation and remodeling (Huertas et al., 2019; Zhao et al., 2024). Oxidative stress occurs when ROS levels exceed the capacity of antioxidant defenses, resulting in endothelial damage, exacerbation of inflammation, and worsening of vascular remodeling (Dorfmüller et al., 2011; Reyes-García et al., 2022).

PAH is increasingly viewed as a metabolic disease, with significant dysregulation in cellular metabolism observed in the lungs, heart, and vasculature. In some respects, these changes resemble cancer-like metabolic profiles (Guignabert et al., 2013; Spiekerkoetter et al., 2019). A hallmark of this metabolic reprogramming is the metabolic shift in PASMC toward glycolysis. Upregulation of pyruvate dehydrogenase kinase 1 (PDK1) and overexpression of the glucose transporter Slc2a1 substantially enhance glycolytic activity in PASMC and EC, promoting cell proliferation and resistance to apoptosis. PDK1 prevents pyruvate from entering mitochondria for oxidative phosphorylation, and cooperates with HIF-1α to drive the Warburg effect (Cuthbertson et al., 2023; Wan et al., 2024; Xu et al., 2021). Dysfunctional mitochondria further exacerbate this process by producing excessive ROS and disrupting calcium homeostasis, amplifying inflammation via the NF-κB pathway and worsening oxidative stress (Reyes-García et al., 2022). This cancer-like metabolic phenotype not only sustains vascular cell proliferation but also represents a rational therapeutic target beyond vasodilation.

**FIGURE 1:**
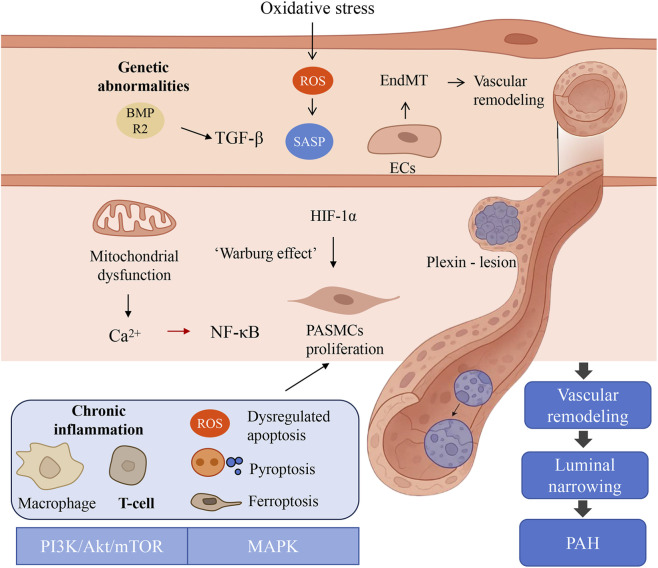
Complex pathogenesis of pulmonary arterial hypertension.

Additionally, dysregulation of multiple signaling pathways exacerbates PAH pathogenesis. Mutations in the bone morphogenetic protein receptor type II (BMPR2) gene, the most common genetic cause of PAH, disrupt transforming growth factor-β (TGF-β) superfamily signaling and lead to unchecked cell proliferation (Awad et al., 2024; Cuthbertson et al., 2023). Overactivation of the PI3K/Akt/mTOR pathway promotes proliferation and survival of PASMC and EC (Shi et al., 2023; Yu et al., 2022), while abnormalities in the mitogen-activated protein kinase (MAPK) pathway affect cell growth, differentiation, and inflammatory responses (Wang X. et al., 2024; Yan et al., 2016). Dysregulation of cell fate decisions, particularly uncontrolled proliferation and differentiation, and imbalance in cell death pathways such as apoptosis, pyroptosis, and ferroptosis, further drives vascular remodeling and inflammation (Figure 1) (Kazmirczak et al., 2024; Li X. et al., 2024; Safaie Qamsari and Stewart, 2024).

Moreover, impairment of the NO–sGC–cGMP pathway, characterized by reduced endothelial nitric oxide synthase (eNOS) expression and diminished NO bioavailability, impairs vasodilation and exacerbates vascular remodeling (Dikalova et al., 2020; Lázár et al., 2020). The interplay among these multifactorial mechanisms explains why single-target therapies are insufficient and underscores the need for treatments that simultaneously target multiple pathological pathways.

### 2.2 Conventional therapeutic targets

As discussed above, PAH pathogenesis is multifactorial, involving vascular remodeling, chronic inflammation, oxidative stress, metabolic reprogramming, endothelial dysfunction, and vasoconstriction. These interconnected mechanisms collectively drive progressive luminal narrowing, increased vascular resistance, and irreversible structural changes in the pulmonary vasculature. Conventional pharmacological therapies primarily target vasoconstriction by modulating vasoactive mediators. Endothelin receptor antagonists, PDE5 inhibitors, and prostacyclin-based drugs restore the balance of ET-1, NO, and prostacyclin signaling, thereby improving pulmonary hemodynamics and clinical symptoms (Bisserier et al., 2020).

However, a major limitation of these therapies is their inability to reverse established pulmonary vascular remodeling or adequately address non-vasoconstrictive pathological processes, including persistent inflammation, oxidative injury, metabolic abnormalities, and endothelial senescence (Table 1). Consequently, current treatments provide symptomatic relief rather than true disease modification, underscoring the inadequacy of single-target vasodilatory strategies in a multifactorial disease such as PAH. Consequently, there is an urgent need to shift treatment toward drugs with pleiotropic properties that can simultaneously modulate these intertwined pathogenic networks. This context establishes the foundation for investigating plant metabolites, which, due to their multi-target mechanisms, represent promising candidates to fill this gap and address the root causes of PAH beyond vasodilation.

**TABLE 1:** PAH pathogenic mechanisms and conventional therapeutic targets.

Pathogenic mechanism	Core pathophysiological role in PAH	Conventional therapeutic targets/Approach	Limitations of conventional approach	References
Vascular remodeling	Excessive proliferation of PASMC, EC, fibroblasts; ECM deposition; luminal narrowing; plexiform lesions	Limited direct anti-proliferative effects (e.g., some prostacyclins, sGC stimulators); sotatercept (BMP/activin pathway) is a recent advance	Often insufficient to reverse established remodeling; many drugs primarily vasodilate rather than directly inhibit cell growth	Evans et al. (2021), Schermuly et al. (2011), Rubin & Naeije, (2023)
Endothelial dysfunction	Loss of anti-proliferative/vasodilatory properties; pro-inflammatory/pro-coagulant phenotype; EndMT; senescence	Indirectly addressed by improving NO/prostacyclin pathways; no direct therapies for EndMT or senescence	Does not fully restore endothelial integrity or reverse dysfunctional phenotype	Culley & Chan, (2022), Evans et al. (2021)
Vasoconstriction	Imbalance of vasoactive mediators (↓NO, ↓prostacyclin, ↑ET-1)	Endothelin receptor antagonists (ERAs), PDE5 inhibitors, sGC stimulators, prostacyclin analogues	Primarily symptomatic relief; does not address underlying structural remodeling or other pathogenic drivers	Davenport et al. (2016), Lázár et al. (2020)
Chronic inflammation	Immune cell infiltration; release of pro-inflammatory cytokines (TNF-α, IL-6, IL-1β); B-cell activation; complement activation	No specific anti-inflammatory drugs for PAH; corticosteroids used in some associated conditions but not primary PAH therapy	Inflammation remains a significant unaddressed driver of disease progression	Huertas et al. (2019), Sanges et al. (2024), Zhao et al. (2024)
Oxidative stress	Imbalance of ROS production and antioxidant defenses; mitochondrial dysfunction; endothelial damage	No specific antioxidant therapies; some drugs may have indirect antioxidant effects	Oxidative damage continues to exacerbate vascular dysfunction and inflammation	Dorfmüller et al. (2011), Reyes-García et al. (2022)
Metabolic reprogramming	“Warburg effect” (aerobic glycolysis); mitochondrial dysfunction; dysregulation of glucose, fatty acid, arginine metabolism	No specific metabolic modulators; some drugs might have minor indirect metabolic impacts	A core driver of PASMC proliferation and disease progression largely unaddressed	Cuthbertson et al. (2023), Xu et al. (2021)
Cell senescence or death	Accumulation of senescent cells; aberrant pyroptosis, apoptosis, ferroptosis	No specific senolytics or modulators of cell death pathways in PAH therapy	Contributes to inflammation and remodeling, representing an emerging therapeutic target.	Kazmirczak et al. (2024), Safaie Qamsari & Stewart, (2024)

## 3 Plant metabolites with potential therapeutic effects on PAH

**FIGURE 2:**
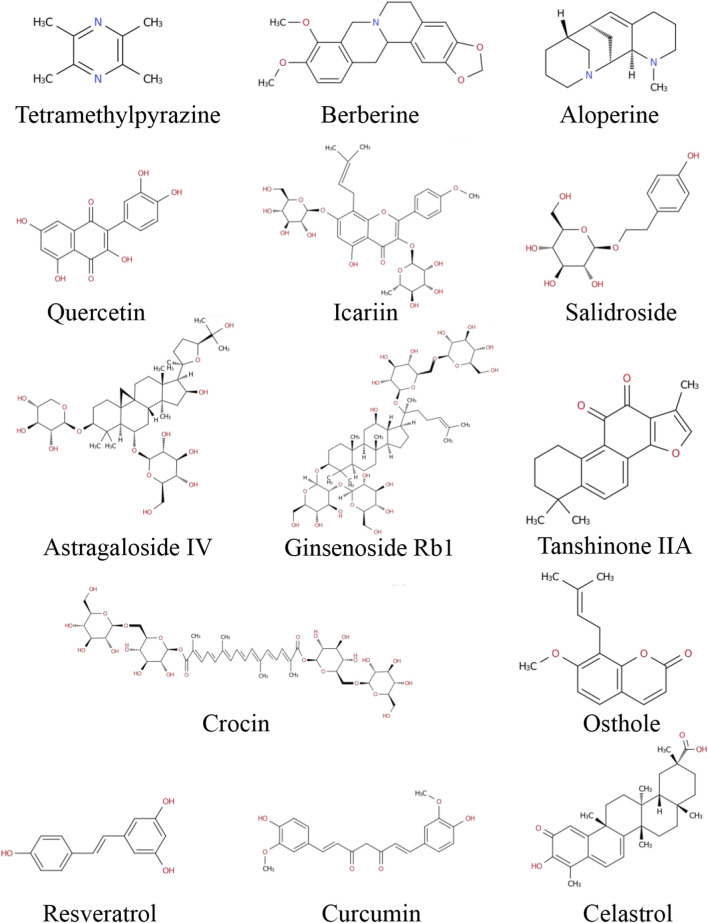
Chemical structures of plant metabolites for PAH treatment.

Plant metabolites exhibit wide structural diversity. These metabolites can be broadly categorized into alkaloids, flavonoids, glycosides, diterpenoids, and other groups based on their chemical structures (Figure 2). They exert anti-PAH effects through multiple molecular mechanisms in a multi-targeted manner (Yu et al., 2022; Zhang et al., 2021). Systematic classification of these metabolites provides a clearer understanding of the relationship between their chemical diversity and pharmacological activities.

### 3.1 Alkaloids

Alkaloids are a class of naturally occurring organic metabolites containing nitrogen atoms and typically exhibit significant biological activities (Bhambhani et al., 2021). Most research on the use of alkaloids for PAH remains preclinical, with animal studies demonstrating that various alkaloids exert anti-PAH effects by regulating cell proliferation, inflammatory responses, and ion channel function.

Tetramethylpyrazine, the primary active component of *Ligusticum chuanxiong Hort*., suggests significant vasodilatory effects in PAH models and clinical trials. It inhibits the proliferation of PASMC and platelet-derived growth factor-BB (PDGF-BB) induced inflammatory responses. It regulates the PI3K/AKT signaling pathway to block cell cycle progression (Huang et al., 2021; Wang et al., 2025b). Clinical studies have demonstrated that oral administration of tetramethylpyrazine (100 mg, three times daily for 16 weeks) improves exercise capacity, as measured by the 6-min walk distance, and enhances heart rate recovery (Chen et al., 2020). However, the available clinical evidence for tetramethylpyrazine is limited to small, single-center, unblinded studies that use surrogate endpoints, such as 6-min walk distance. Robust multicenter trials with hemodynamic and long-term outcome measures are still lacking.

Berberine, found in medicinal plants such as *Coptis chinensis Franc*h., exhibits anti-inflammatory, antioxidant, and cardiovascular-protective properties (Luo et al., 2021). It alleviates pulmonary vascular remodeling by inhibiting abnormal PASMC proliferation, migration, and resistance to apoptosis. Key mechanisms involve the regulation of protein phosphatase 2A (PP2A) and BMP/TGF-β signaling pathways, as demonstrated in both *in vivo* and *in vitro* studies (Chen M. et al., 2019; Luo et al., 2018). Recent studies have further shown that berberine (30 mg/kg), administered daily for 3 weeks, reduces pulmonary inflammatory factors and oxidative stress. In combination with other metabolites, such as quercetin, it improves pathological indicators in MCT-induced PAH models (Beik et al., 2023; Kordestani et al., 2024). Although berberine is not classified as a classic PAINS, its intrinsic fluorescence and pleiotropic bioactivity may confound *in vitro* assays. Direct target engagement and clinically relevant exposure in PAH, therefore, require further validation. Meanwhile, no clinical studies on berberine for PAH have been conducted to date, and further research is required to evaluate its clinical efficacy and potential toxicity.

Aloperine is a quinolizidine alkaloid extracted from *Sophora flavescens Aiton*. It inhibits PDGF-BB-induced PASMC proliferation by blocking cell cycle progression and promoting apoptosis *in vitro*. Oral administration of aloperine (25, 50, or 100 mg/kg/day for 21 days) reduces inflammation in MCT-induced PAH by negatively regulating the NF-κB signaling pathway (Chang et al., 2019; Li, 2019; Wang et al., 2025c). Currently, researchers have developed aloperine-loaded nanostructured lipid carriers (NLCs) to enhance its bioavailability, providing a basis for its potential clinical application in PAH treatment (Liu H. et al., 2025) In addition, sanguinarine improves PAH by reducing PASMC proliferation and resistance to apoptosis through the downregulation of selenoprotein P (SeP) expression, suggesting SeP as a potential novel therapeutic target (Kikuchi et al., 2018).

Overall, the evidence for alkaloids in PAH is largely derived from *in vitro* experiments and rodent models, with tetramethylpyrazine representing the only metabolite supported by limited clinical data. For most alkaloids, the lack of large-scale, well-controlled clinical trials and comprehensive pharmacokinetic and long-term safety assessments currently limits firm conclusions regarding their clinical relevance.

### 3.2 Flavonoids

Flavonoids are widely reported to exert anti-inflammatory and antioxidant effects in PAH models; however, many members of this class are also known for promiscuous bioactivity and potential PAINS-related assay interference, necessitating cautious interpretation of mechanistic claims.

Quercetin is a widely studied flavonol that has been reported to exert anti-inflammatory and antioxidant effects in experimental PAH models. Quercetin (5 mg/kg/d) administered for 14 days improves the hemodynamic changes, RVH (right ventricular hypertrophy), and pulmonary vascular remodeling in MCT-induced PAH rats. It also inhibits the proliferation, migration, and phenotypic transformation of PASMC by regulating the TGF-β1/Smad2/Smad3 pathway in PDGF-BB-induced cellular models (Gao et al., 2024). In addition, quercetin reduces the inflammatory response and arteriolar wall thickness in the MCT-induced PAH model and acts synergistically with berberine to improve pathological outcomes (Kordestani et al., 2024; Rajabi et al., 2020). Although quercetin is a widely studied flavonoid that has been reported to have anti-inflammatory and antioxidant effects in PAH models, it must be interpreted with extreme caution. As a prototypical polyphenolic PAINS compound, its *in vitro* activities (e.g., inhibition of PASMC proliferation at 10–50 μM) are often observed at concentrations exceeding pharmacologically relevant free plasma levels and may result from assay interference (e.g., redox cycling, protein aggregation) rather than specific target modulation (Bolz et al., 2021; Magalhães et al., 2021). Although animal studies show amelioration of MCT-PAH, causal links to specific molecular targets remain unproven, and its clinical relevance is undetermined.

Icariin is the main active flavonoid glycoside of *Epimedium brevicornu Maxim* and has various pharmacological effects, including anti-inflammatory, antioxidant, and immune-regulatory effects (Song et al., 2025). Studies have shown that icariin (20, 40, and 80 mg/kg/day) significantly alleviates PAH by enhancing NO/cyclic guanosine monophosphate (cGMP) signaling. It achieves this by upregulating eNOS gene expression and downregulating PDE5 gene expression, thereby increasing NO and cGMP levels, promoting pulmonary vasodilation, and ameliorating MCT-induced PAH (Li et al., 2016). Icariin exerts effects in MCT-induced PAH primarily via potential PDE5 modulation, but direct enzyme inhibition and target engagement have not been confirmed with orthogonal assays.

Other flavonoids also demonstrate therapeutic potential. Luteolin attenuates MCT-induced pulmonary vascular remodeling and RVH in rats and inhibits PASMC proliferation and migration by suppressing the Hippo-YAP/PI3K/AKT signaling pathway (Zuo et al., 2021). Isorhamnetin inhibits PASMC proliferation and improves hemodynamics and oxidative stress levels in PAH rats by regulating the BMP signaling pathway and the p-c-src/NOX1 pathway (Chang et al., 2020; Chen F. et al., 2024). Baicalin suppresses hypoxia-induced PASMC proliferation, migration, and resistance to apoptosis, and mitigates EndMT by upregulating the adenosine A2a receptor (A2aR) and modulating the NF-κB/BMP signaling pathway (Huang et al., 2018; Zhang et al., 2017). Baicalin’s multi-pathway effects may reflect upstream regulatory roles or non-specific stress responses, and its direct target in PAH requires further validation with rescue experiments. Rutin inhibits ferroptosis in PAH by interacting with protein kinase Cα (PKCα), suggesting its potential role in regulating mitochondrial metabolism (Che et al., 2024).

Notably, several flavonoids discussed above (e.g., quercetin, luteolin) are frequently reported as broadly bioactive *in vitro*. They may display PAINS-like or promiscuous assay behavior, particularly when activity is concluded from a single biochemical/cell-based readout at high micromolar concentrations. Therefore, mechanistic claims based mainly on *in vitro* inhibition should be interpreted cautiously unless supported by dose–response relationships, appropriate counterscreens, orthogonal assays, and evidence of target engagement *in vivo* (Bolz et al., 2021; Magalhães et al., 2021).

### 3.3 Glycosides

Glycoside metabolites are a class of natural products formed by connecting glycosyl groups with aglycones via glycosidic bonds. They generally exhibit good water solubility and possess various biological activities, including anti-inflammatory, antioxidant, and antitumor properties.

Salidroside is a natural glycoside compound extracted from the plant Rhodiola rosea L. It possesses multiple biological activities, including antioxidant, anti-inflammatory, anti-fibrotic, cardiovascular protection, anti-fatigue, and anti-aging effects (Liang et al., 2024). Research demonstrated that salidroside (2, 8, and 32 mg/kg/d) significantly protects against hypoxia-induced PAH by inhibiting the AhR/NF-κB pathway and activating the Nrf2/HO-1 pathway, alleviating oxidative stress in pulmonary artery EC (Lei et al., 2024). Additionally, administration of salidroside (25 and 50 mg/kg/d) effectively attenuates pulmonary vascular remodeling, oxidative stress, and inflammation. It enhances NO synthesis and bioavailability by modulating the arginine metabolic pathway in MCT-induced PAH rats. (Li J. et al., 2024).

Astragaloside IV is one of the primary active metabolites of *Astragalus membranaceus (Fisch.) Bunge* (Yao et al., 2024). Administration of astragaloside IV (10 and 30 mg/kg/day for 21 days) effectively attenuates pulmonary vascular remodeling, RVH, and TNF expression in MCT-induced PAH rats. Astragaloside IV significantly reduced hypoxia-induced increases in HIF-1α and VEGF protein levels in human pulmonary artery endothelial cells (Jin et al., 2020; Xi et al., 2023; Yao et al., 2020). It also mitigates cell pyroptosis and fibrosis progression in MCT-induced PAH rats, demonstrating its potential role in the prevention and treatment of PAH (Xi et al., 2023).

Ginsenoside Rb1 is one of the primary active metabolites *of Panax ginseng C. A. Mey*. Research confirmed that Ginsenoside Rb1 (30 mg/kg/d for 21 days) reduces MCT-induced expression of STIM1, TRPC1, and TRPC4, as well as calcium influx related to store-operated calcium entry (SOCE) and pulmonary artery constriction, thereby improving hemodynamic and vascular remodeling indicators in PAH and ultimately achieving the goal of alleviating MCT-induced PAH (Wang et al., 2020). Additionally, it reverses hypoxia-induced EndMT and inflammation by regulating the CCN1 pathway (Tang et al., 2023).

Nevertheless, most studies on glycoside metabolites focus on short-term efficacy in rodent models, and systematic evaluations of exposure–response relationships, chronic dosing, and long-term toxicity remain largely unavailable.

### 3.4 Diterpenes

Tanshinone IIA is the primary lipid-soluble active component of *Salvia miltiorrhiza Bunge*. Previous studies have demonstrated that tanshinone IIA can upregulate the protein levels of p27, vascular smooth muscle protein kinase G (PKG), and PPAR-γ, therefore inhibiting hypoxia-induced pulmonary artery wall thickening and PASMC proliferation, significantly reducing mPAP (Ding Z. et al., 2025). It also increases pulmonary blood flow, inhibits pulmonary small-vessel remodeling, protects the vascular endothelium, and reduces platelet aggregation in pulmonary vessels (Ding Z. et al., 2025). Furthermore, its derivative, sodium tanshinone IIA sulfonate (STS), reduces the expression of pro-inflammatory cytokines, upregulates the expression of BMPR2, and enhances the phosphorylation of Smad1/5/9 to exert anti-apoptotic effects in hypoxia-induced PAH (Wang et al., 2022). STS has successfully entered clinical trials for PAH, demonstrating its potential to reduce pulmonary arterial pressure and improve exercise capacity in patients (Shang et al., 2012; Xue Z. et al., 2021). The translational development of STS in treating PAH has already been conducted in exploratory clinical trials in China (ChiCTR-IPR-15006669).

STS represents the advanced candidate, supported by clinical evidence for efficacy in PAH, whereas most plant metabolites remain at the preclinical stage. This disparity underscores the importance of formulation and pharmacokinetic optimization, as well as rigorous clinical validation, to translate promising experimental findings into therapeutic applications.

### 3.5 Others

In addition to the main categories mentioned above, many other structural types of plant metabolites suggest significant pharmacological activity against PAH.

Crocin is a glycosylated apocarotenoid derived from *Crocus sativus L.*, and has been reported to exert anti-remodeling effects in experimental PAH models. Crocin (50 mg/kg every 3d) alleviates inflammatory responses, improves pulmonary artery remodeling, and reduces right ventricular systolic pressure and mean pulmonary artery pressure (mPAP), thereby ameliorating MCT-PAH, as well as inhibiting TGF-β1-induced myofibroblast activation to alleviate hypoxia-induced PAH in mice (Deng et al., 2024; Sheng et al., 2021). Integration of scRNA-seq with *in vitro* and *in vivo* analyses shows that crocin inhibits PASMC proliferation by suppressing neutrophil migration and activation through targeting HCK, ultimately alleviating hypoxia-induced pulmonary vascular remodeling and pulmonary arterial hypertension (Sheng et al., 2025).

Osthole is a pyranocoumarin compound extracted from *Cnidium monnieri (L.)* Cuss. Proteomic studies have revealed that osthole (80 mg/kg for 28 days) can significantly restore the expression of multiple differentially expressed proteins involved in the ribosome, oxidative phosphorylation, and complement/coagulation cascade pathways during the progression of PAH in MCT-induced rats, suggesting its potential as a novel multi-target, multi-pathway therapeutic candidate for PAH (Yao et al., 2018).

Resveratrol is a polyphenolic compound with powerful antioxidant, anti-inflammatory, and cardiovascular protective effects. Resveratrol (5, 15, 30, 50 µM) inhibits the proliferation and migration of PASMC via the PI3K/AKT signaling pathway *in vitro*, activates silent information regulator 1 (SIRT1) to reverse PAH, and alleviates oxidative stress and inflammatory responses in hypoxia-induced PAH rats (Guan et al., 2017; Yu et al., 2017; Xu et al., 2016). However, these *in vitro* concentrations (up to 50 µM) may exceed pharmacokinetically achievable free plasma or lung exposure after conventional oral dosing, and polyphenols may show assay interference; therefore, the translational relevance of single-assay *in vitro* findings requires confirmation by orthogonal target engagement assays and *in vivo* exposure–response analyses (Bolz et al., 2021; Magalhães et al., 2021). Meanwhile, the low oral bioavailability of resveratrol (<10%) poses a challenge for clinical application, which prompts researchers to develop lung-targeted nanoparticles as inhalation carriers to enhance its bioavailability (Li et al., 2020).

Curcumin is a fat-soluble phenolic pigment extracted from *Curcuma longa L.* Research indicates that curcumin provides vascular protection against arterial hypertension by inhibiting vasoconstriction, blocking PASMC proliferation and migration, and improving endothelial dysfunction (Amin et al., 2021). Furthermore, curcumin nanoparticles (50 mg/kg for 7 days) promote apoptosis in PASMC, thereby reducing mPAP and reversing pulmonary arterial remodeling (Rice et al., 2016). Curcumin is frequently cited for its multi-target potential. However, it is a textbook example of a PAINS (Bolz et al., 2021; Magalhães et al., 2021). Its apparent effects in cellular assays, including anti-proliferation and anti-inflammation, are highly likely to be artifacts due to its reactivity, instability, and propensity to generate oxidative byproducts. No study to date has conclusively demonstrated direct, specific target engagement of curcumin in PAH pathophysiology. Its notoriously poor oral bioavailability further renders most *in vivo* findings pharmacologically uninterpretable. Therefore, curcumin should not be considered a viable lead compound for PAH drug discovery without a revolutionary delivery system and unequivocal target validation using PAINS-aware orthogonal assays.

Celastrol is a pentacyclic triterpenoid compound, and administration of celastrol (1 mg/kg per 48 h) reduces levels of Bsg, CyPA, and inflammatory cytokines in the heart and lungs of hypoxia-induced PAH mice and SU5416/hypoxia-induced PAH rats, while improving right ventricular systolic pressure, hypertrophy, fibrosis, and dysfunction (Kurosawa et al., 2021). Additionally, celastrol improves hypoxia-induced PAH by modulating the PDE5-cGMP-PKG signaling pathway and reduces right ventricular systolic pressure, hypertrophy, and dysfunction (Tan et al., 2025).

Moreover, network pharmacology analysis indicates that chlorogenic acid has potential therapeutic effects on PAH by targeting key hub targets, including tumor protein p53 (TP53), HIF-1α, and interleukin-1 beta (IL-1β) (Santos-Álvarez et al., 2024). However, such *in silico* predictions do not demonstrate target engagement or efficacy and require experimental validation with appropriate controls and exposure-relevant dosing. Pterostilbene alleviates PAH by inhibiting EndMT, reducing high mobility group AT-hook 2 (HMGA2) expression, and restoring von Willebrand factor (Wang J. et al., 2025). Oroxylin A inhibits the progression of PAH and pulmonary arterial remodeling by suppressing the Warburg effect to improve aerobic glycolysis (Wang et al., 2024c). Usnic acid, similar to synthetic inhibitors such as sildenafil, has the potential to act as a PDE5 inhibitor, suggesting its promising application in the synergistic treatment of PAH (Đorović Jovanović et al., 2025). Usnic acid shows potential PDE5 inhibition *in silico* but is a potential PAINS with known hepatotoxicity, necessitating rigorous safety evaluation (Bolz et al., 2021; Magalhães et al., 2021).

Metabolites in this heterogeneous category provide valuable mechanistic hypotheses and expand the landscape of potential PAH targets. However, some conclusions are derived from network pharmacology, molecular docking, or isolated *in vitro* assays. Such *in silico* and exploratory approaches should be regarded as hypothesis-generating rather than confirmatory, and require rigorous experimental validation with appropriate controls and exposure-relevant models.

In summary, the diverse plant metabolites demonstrate multi-target capabilities by targeting the multifactorial pathogenesis of PAH through antiproliferative, anti-inflammatory, antioxidant, and metabolic regulatory mechanisms. This multifunctionality offers unique therapeutic advantages over traditional single-target drugs when addressing the complex nature of PAH. However, a significant gap remains between preclinical research and clinical translation. Current evidence heavily relies on rodent models (MCT or hypoxia), which may not fully replicate human pathologies. Moreover, many candidates face substantial challenges, including low bioavailability (e.g., curcumin, resveratrol) and unclear toxicological profiles. Although a few metabolites, such as tetramethylpyrazine and sodium tanshinone IIA sulfonate, have advanced to clinical trials, most remain confined to preclinical research. Rigorous validation in large-scale clinical studies is essential to confirm their safety and efficacy in human subjects.

## 4 Multi-pathway mechanisms of plant metabolites against PAH

### 4.1 Plant metabolites targeting pulmonary vascular tone

**FIGURE 3:**
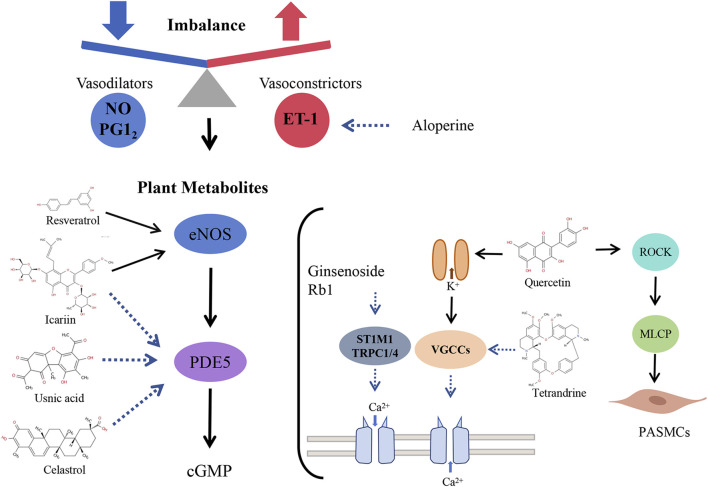
Plant metabolites regulate pulmonary arterial vascular tone by balancing vascular active substances.

A core pathophysiological hallmark of PAH is the dysregulation of pulmonary vascular tone, resulting from a profound imbalance between vasodilatory and vasoconstrictive forces. This imbalance, driven largely by endothelial dysfunction, leads to reduced bioavailability of vasodilators such as NO and prostacyclin, accompanied by excessive production of vasoconstrictors including endothelin-1 (ET-1). Increasing evidence indicates that plant metabolites act as multi-target modulators that restore vascular homeostasis by simultaneously regulating multiple signaling pathways, thereby alleviating pathological vasoconstriction and reducing pulmonary arterial pressure (Figure 3) (Martin de Miguel et al., 2023; Zolty, 2023).

#### 4.1.1 Restoration of vasoactive mediator balance

The NO–sGC–cGMP signaling cascade plays a central role in pulmonary vasodilation but is frequently impaired in PAH due to eNOS downregulation and PDE5 upregulation. Several plant metabolites have been shown to correct this imbalance. For example, icariin exerts dual regulatory effects by upregulating eNOS while downregulating PDE5, thereby increasing NO bioavailability and intracellular cGMP levels, ultimately reducing pulmonary arterial pressure (Li et al., 2016).

Similarly, resveratrol enhances eNOS activity and NO production, whereas usnic acid has been proposed as a potential natural PDE5 inhibitor that stabilizes cGMP levels (Li et al., 2019; Đorović Jovanović et al., 2025). However, the PDE5-inhibitory activity of usnic acid is currently supported only by *in silico* evidence, and it is also classified as a potential PAINS with known hepatotoxicity, necessitating rigorous experimental validation of its safety and efficacy (Bolz et al., 2021; Magalhães et al., 2021).

In addition, celastrol has been reported to exert anti-proliferative effects in hypoxia-induced PAH through modulation of the PDE5–cGMP–PKG axis, an effect that can be reversed by PDE5 overexpression, further supporting the involvement of this pathway (Tan et al., 2025). Beyond NO signaling, plant metabolites also counteract excessive vasoconstrictor activity. Resveratrol and aloperine suppress ET-1 synthesis and expression in monocrotaline (MCT)-induced PAH models, thereby contributing to restoration of vascular tone homeostasis (Li, 2019; Li et al., 2019).

#### 4.1.2 Modulation of ion channels

Intracellular calcium concentration ([Ca^2+^]ᵢ) governs both the contractile and proliferative phenotypes of PASMC and represents a major driver of pulmonary vasoconstriction. Plant metabolites exert beneficial effects on PAH by targeting key Ca^2+^ influx pathways, thereby reducing intracellular calcium levels.

Alkaloids such as tetrandrine act as direct antagonists of L-type voltage-gated calcium channels (VGCCs), thereby inhibiting Ca^2+^ entry and attenuating vasoconstriction (Xiang et al., 2018). The store-operated calcium entry (SOCE) pathway, mediated by stromal interaction molecule (STIM) and transient receptor potential canonical (TRPC) channels, is a critical source of pathological Ca^2+^ influx in PAH. Ginsenoside Rb1 improves MCT-induced PAH by downregulating STIM1, TRPC1, and TRPC4 expression, suppressing SOCE and subsequent pulmonary vasoconstriction (Wang et al., 2020). Similarly, chrysin attenuates chronic hypoxia-induced PAH by inhibiting SOCE, lowering [Ca^2+^]ᵢ, and suppressing hypoxia-inducible factors such as HIF-1α and TRPC1/6 (Dong et al., 2019; Dong et al., 2020; Wang et al., 2019).

Potassium (K^+^) channels in PASMC are functionally coupled to Ca^2+^ influx. The opening of these K^+^ channels facilitates K^+^ efflux, inducing membrane hyperpolarization that inactivates VGCCs, limits Ca^2+^ influx, and promotes vasodilation. Tanshinone IIA reverses hypoxia-induced downregulation of Kv1.5 and Kv2.1 channels, restoring K^+^ currents and reducing pulmonary vascular tone (Zhang et al., 2021). Other plant metabolites, including quercetin and ginsenosides, also exert vasodilatory effects through K^+^ channel-dependent mechanisms (Rajabi et al., 2020; Wang X. et al., 2024; Zeng et al., 2023).

#### 4.1.3 Direct targeting of the vascular smooth muscle contractile apparatus

Beyond receptor-mediated signaling and ion channel modulation, direct inhibition of the vascular smooth muscle contractile machinery represents an additional therapeutic strategy. The RhoA/Rho-associated protein kinase (ROCK) pathway promotes vasoconstriction by inhibiting myosin light chain phosphatase (MLCP). Quercetin has been identified as a functional ROCK inhibitor (mechanism not directly validated) capable of inducing vasodilation independently of changes in [Ca^2+^]ᵢ (Wang et al., 2021).

Nevertheless, most evidence supporting these mechanisms is derived from marker-based pathway analyses. Causal validation through genetic manipulation or pharmacological rescue experiments remains limited, underscoring the need for more rigorous mechanistic studies.

### 4.2 Plant metabolites modulating vascular remodeling and cell proliferation

Pulmonary vascular remodeling, characterized by excessive proliferation of PASMC and EC, along with ECM accumulation, is a central determinant of increased pulmonary vascular resistance in PAH (Guignabert et al., 2024; Shimoda, 2020). Accumulating data indicate that plant metabolites possess substantial potential to arrest or reverse this pathological process.

#### 4.2.1 Inhibition of proliferative signaling pathways

Abnormal activation of growth factor signaling is central to remodeling. Loss of BMPR2 is a genetic hallmark of PAH. Mechanistically, baicalin is reported to exert anti-proliferative effects in PASMC via the BMPR2/Smad and A2AR/PI3K/AKT pathways, though the direct target engagement and causal relationship remain to be validated (Table 2); it also induces cell cycle arrest by inhibiting HIF-1α, stabilizing p27, and blocking platelet-derived growth factor receptor (PDGFRβ) downstream ERK1/2 activation (Huang et al., 2017).

**TABLE 2:** Evidence level and PAINS risk of representative plant metabolites investigated in PAH.

Plant metabolite	Main reported mechanisms in PAH	Evidence level	PAINS/Translational risk	Key references
Tetramethylpyrazine	Vasodilation; inhibition of PASMC proliferation; anti-inflammatory effects via PI3K/AKT signaling	Limited clinical + animal	Low–moderate (relatively clear PK; small clinical trials)	Chen et al., 2020; Huang et al., 2021
Berberine	Anti-proliferative and anti-inflammatory effects via PP2A and BMP/TGF-β signaling	Animal + *in vitro*	Moderate (fluorescence interference; pleiotropic activity)	Luo et al., 2018; Chen et al., 2019a; Beik et al., 2023
Aloperine	Inhibition of PASMC proliferation; NF-κB suppression	Animal + *in vitro*	Moderate (limited PK and safety data)	Chang et al., 2019; Wang et al., 2025b
Quercetin	Anti-oxidative stress; inhibition of PASMC proliferation via TGF-β/Smad	Animal + *in vitro*	High (polyphenol PAINS; high-dose *in vitro* use)	Rajabi et al., 2020; Gao et al., 2024
Icariin	Enhancement of NO–cGMP signaling; PDE5 modulation	Animal	Moderate (target engagement not fully validated)	Li et al. (2016)
Luteolin	Inhibition of PASMC proliferation via hippo-yap/PI3K/AKT	Animal + *in vitro*	High (PAINS-like behavior *in vitro*)	Zuo et al. (2021)
Baicalin	Anti-proliferative, anti-inflammatory; modulation of NF-κB and BMP signaling	Animal + *in vitro*	High (multi-pathway effects; indirect target evidence)	Zhang et al., 2017; Huang et al., 2018
Salidroside	Anti-oxidative stress via Nrf2/HO-1; anti-inflammatory via AhR/NF-κB	Animal	Moderate (short-term studies; limited PK data)	Lei et al., 2024; Li et al., 2024a
Astragaloside IV	Suppression of HIF-1α/VEGF; anti-inflammatory effects	Animal	Moderate (limited chronic toxicity data)	Jin et al., 2020; Xi et al., 2023
Ginsenoside Rb1	Inhibition of SOCE via STIM1/TRPC; reversal of EndMT	Animal	Low–moderate (complex PK; limited lung exposure data)	Wang et al., 2020; Tang et al., 2023
Crocin	Anti-inflammatory; inhibition of PASMC proliferation; immune modulation	Animal	Moderate (classification issues; exposure uncertainty)	Sheng et al., 2021; Deng et al., 2024
Tanshinone IIA sulfonate (STS)	Anti-proliferative; BMPR2/Smad activation; endothelial protection	Clinical + animal	Low (clinical formulation available)	Shang et al., 2012; Wang et al., 2022; Xue et al., 2021b
Resveratrol	Anti-oxidative stress; SIRT1 activation; metabolic regulation	Animal + *in vitro*	High (PAINS; poor bioavailability)	Xu et al., 2016; Yu et al., 2017; Li et al., 2020
Curcumin	Anti-proliferative; apoptosis induction; metabolic regulation	Animal + *in vitro*	Very high (classic PAINS; poor PK)	Rice et al., 2016; Magalhães et al., 2021
Celastrol	Anti-inflammatory; PDE5-cGMP-PKG modulation	Animal	Moderate–high (narrow therapeutic window)	Kurosawa et al., 2021; Tan et al., 2025

Evidence level reflects the highest level of experimental support reported to date. PAINS/translational risk assessment considers assay promiscuity, pharmacokinetic limitations, safety concerns, and clinical validation status.

Baicalein downregulates the ET-1 system by blocking the AKT/ERK/GSK3β/β-catenin pathway, limits calcium influx by inhibiting the PKCα/TRPC1 axis, and suppresses PASMC proliferation as a potent ROCK inhibitor. Furthermore, Luteolin inhibits PASMC proliferation and migration in a concentration-dependent manner by downregulating large tumor suppressor 1 (LATS1) and Yes-associated protein (YAP), thereby restricting nuclear YAP translocation and AKT phosphorylation (Zuo et al., 2021). Forsythiaside B exerts protective effects against PAH by inhibiting NF-κB signaling and reversing excessive proliferation and migration of PASMC (Liu J. et al., 2024). Irisin modulates the ubiquitination status of Enolase 1 via the E3 ligase NEDD4, thereby inhibiting PDGF-induced proliferation of PASMC (Sun N et al., 2025).

#### 4.2.2 Induction of apoptosis and inhibition of senescence

Restoring apoptosis in hyper-proliferative PASMC is a key therapeutic goal. Puerarin exhibits potent pro-apoptotic effects on remodeled pulmonary vessels. Mechanistic studies reveal that puerarin significantly inhibits the phosphorylation of PI3K and Akt, thereby blocking the survival signaling in hypoxic PASMC and triggering mitochondrial-dependent apoptosis (Chen et al., 2012; Zhang et al., 2019). Targeting cellular senescence is also emerging. Ginsenoside Rg1 ameliorates vascular remodeling by inhibiting the cGAS/STING pathway, reducing senescence-associated secretory phenotype (SASP) factors (Ding R. et al., 2025). These findings highlight the potential of these metabolites to restore the balance between cell proliferation and death in the pulmonary vasculature.

#### 4.2.3 Regulation of extracellular matrix (ECM) and EndMT

Emerging evidence suggests that targeting EndMT is a viable strategy to limit aberrant ECM production. By downregulating HMGA2, pterostilbene inhibits the TGF-β1/Smad2/3 signaling axis, thereby suppressing EndMT. This inhibition significantly reduces the transdifferentiation of EC into ECM-secreting myofibroblasts, ultimately diminishing ECM in the pulmonary arterial wall (Wang J. et al., 2025). Notably, baicalin and baicalein act synergistically to reverse EndMT, restore endothelial homeostasis, and alleviate ECM abnormalities (Cui et al., 2022).

Pulmonary vascular remodeling elevates pulmonary arterial pressure, directly increasing the afterload on the right ventricle (RV) and ultimately leading to right ventricular hypertrophy and failure. Plant metabolites indirectly alleviate RV burden and preserve function by improving pulmonary vascular remodeling. For instance, tetramethylpyrazine, traditionally used in cardiovascular therapeutics, has demonstrated efficacy in both animal models and clinical studies, where it reduces RV load and ameliorates PAH by inhibiting vascular remodeling (Chen et al., 2020).

**FIGURE 4:**
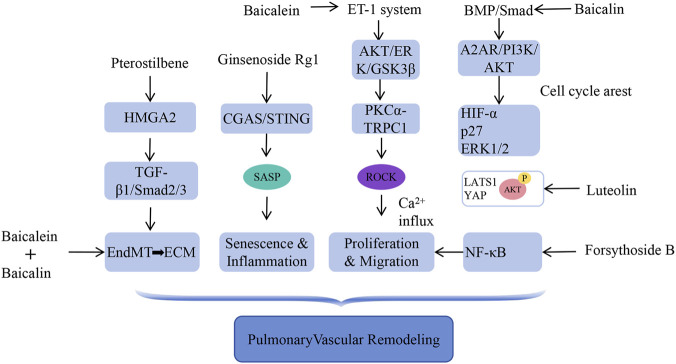
Plant metabolites can improve pulmonary arterial hypertension by interfering with key signaling pathways that regulate cell growth and vascular remodeling.

Collectively, plant metabolites modulate key signaling pathways governing cell growth and remodeling (Figure 4). These diverse mechanisms highlight the capacity of plant metabolites to address vascular remodeling through multifaceted approaches, potentially offering broader therapeutic solutions than existing strategies.

### 4.3 Plant metabolites modulating inflammation and oxidative stress

**FIGURE 5:**
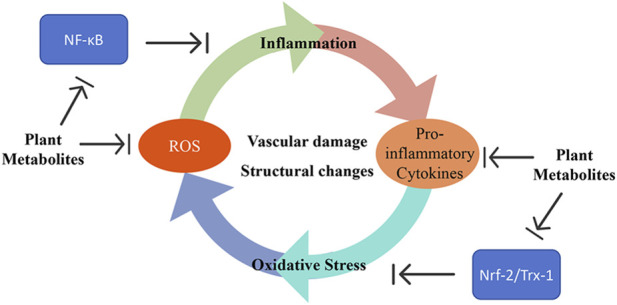
Plant metabolites can break the vicious cycle of vascular damage and structural alterations that lead to pulmonary arterial hypertension due to their potent anti-inflammatory and antioxidant capabilities.

Persistent inflammation and oxidative stress are tightly interconnected in PAH, forming a self-perpetuating vicious cycle that drives vascular injury and structural remodeling (Huertas et al., 2019; Reyes-García et al., 2022). Accumulating evidence suggests that plant metabolites exhibit potent anti-inflammatory and antioxidant properties, positioning them as promising candidates to disrupt this pathological interplay (Figure 5).

#### 4.3.1 Suppression of inflammatory cascades

The infiltration of macrophages and the release of cytokines are the major drivers of inflammatory responses. Andrographolide functions as a potent NF-κB inhibitor, blocking the nuclear translocation of p65 and subsequently reducing the expression of pro-inflammatory cytokines such as TNF-α and IL-6 in MCT-induced PAH rats (Nie et al., 2021). Baicalin attenuates inflammation by modulating NF-κB signaling and upregulating its endogenous inhibitor I-κBα (Zhang et al., 2017). Many plant metabolites, such as resveratrol, berberine, astragaloside IV, isoliquiritigenin, and tetramethylpyrazine, have been shown to reduce the production and release of pro-inflammatory cytokines and chemokines, thereby alleviating inflammatory infiltration (Beik et al., 2023; Jin et al., 2019; Jin et al., 2020; Huang et al., 2021; Kurosawa et al., 2021; Li et al., 2019). Notably, rutin introduces a novel mechanism by interacting with PKCα to inhibit ferroptosis-associated inflammation (Che et al., 2024).

#### 4.3.2 Restoration of redox homeostasis

Oxidative stress in PAH arises from an imbalance between reactive oxygen species (ROS) production and antioxidant defense capacity. Excessive ROS accumulation exacerbates PASMC proliferation, endothelial dysfunction, and inflammatory activation. Plant metabolites restore redox homeostasis by activating endogenous antioxidant pathways and directly scavenging free radicals.

Resveratrol attenuates hypoxia-induced PAH by activating the Nrf2/Trx-1 antioxidant pathway and suppressing ROS generation (Xu et al., 2016). Isorhamnetin reduces oxidative stress by upregulating Nrf2 expression, enhancing antioxidant defenses, and inhibiting NOX1 activity (Chen F. et al., 2024). Quercetin and berberine similarly alleviate oxidative and inflammatory stress in PAH models through multi-pathway regulation, restoring redox balance and enhancing antioxidant enzyme activity (Beik et al., 2023; Rajabi et al., 2020). Notably, the phenolic hydroxyl groups of resveratrol and quercetin confer strong ROS-scavenging capacity, contributing to their antioxidant efficacy (Zhang et al., 2024).

### 4.4 Plant metabolites modulating metabolic reprogramming in PAH

**FIGURE 6:**
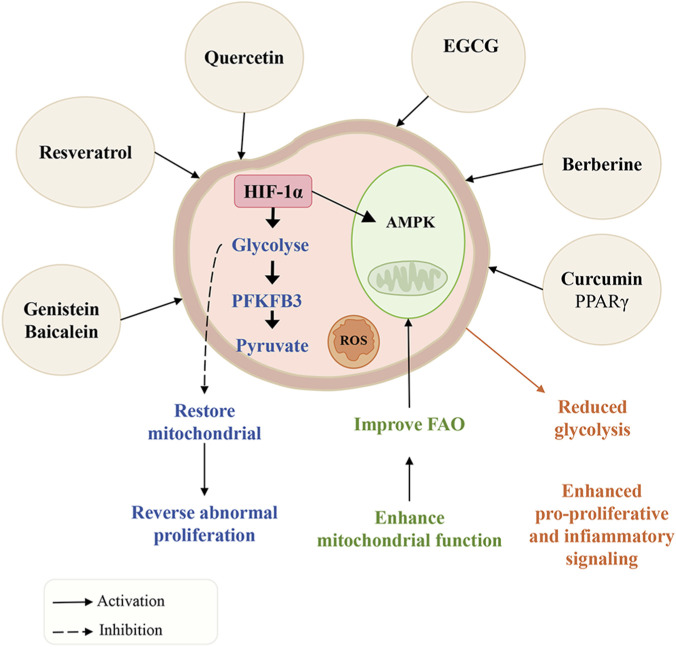
Plant metabolites exert anti-pulmonary arterial hypertension effects by targeting key nodes in metabolic reprogramming to restore metabolic homeostasis.

Recent studies have found that one characteristic of PAH is cellular metabolic reprogramming. This reprogramming leads to excessive proliferation, resistance to apoptosis, and an inflammatory phenotype in vascular cells. These metabolic changes share some similarities with cancer, including disorders in glucose, fatty acid, and amino acid metabolism, as well as mitochondrial dysfunction. All these disorders together create a metabolic state that is conducive to vascular remodeling (Pei et al., 2025; Yi et al., 2021; Zhao et al., 2023). Plant metabolites have demonstrated promising therapeutic potential by targeting key nodes in these dysregulated pathways to restore metabolic homeostasis (Figure 6).

#### 4.4.1 Reversing the warburg effect

A notable feature of metabolic reprogramming in PAH is the shift of glucose metabolism towards glycolysis, known as the Warburg effect. Plant metabolites target key glycolytic enzymes to shift energy metabolism from glycolysis. Resveratrol, curcumin, and quercetin (all classified as PAINS) are associated with reduced HIF-1α in PAH models, which may reflect a general anti-hypoxic response rather than a specific target effect, given the lack of *in vivo* exposure data (Bolz et al., 2021; Magalhães et al., 2021; Xu et al., 2016). Without matching *in vivo* exposure data, reductions in HIF-1α expression should be interpreted cautiously, as they may represent secondary anti-hypoxic or cytotoxic effects rather than specific metabolic targeting. Genistein and baicalein directly act on 6-phosphofructose-2-kinase/fructose-2,6-bisphosphatase 3 (PFKFB3), which is a key activator of the glycolysis metabolic pathway, suppressing the glycolytic activity in hyper-proliferative PASMC (Chen Y. et al., 2019).

#### 4.4.2 Enhancing mitochondrial function and FAO

Restoring mitochondrial health is essential for metabolic flexibility. Furthermore, berberine stimulates adenosine monophosphate-activated protein kinase (AMPK) activity, prompting cells to use oxidative phosphorylation rather than glycolysis for energy production (Chen T. et al., 2024). In addition, plant metabolites also repair defective fatty acid oxidation (FAO) and improve mitochondrial function. For instance, curcumin increases the expression of peroxisome proliferator-activated receptor γ (PPARγ). At the same time, resveratrol and berberine activate the SIRT1/PGC-1α pathway, thereby promoting mitochondrial biogenesis and enhancing fatty acid oxidation (Hu et al., 2024). Moreover, Epigallocatechin gallate (EGCG) exerts its effects on PAH by protecting mitochondrial structure and reducing ROS production (Zhu et al., 2017).

Metabolic reprogramming is closely related to vascular remodeling and systemic inflammation. Intermediate products generated by the glycolytic pathway and ROS stimulate the HIF-1α and mTOR pathways, which promote cell growth, and activate the NF-κB pathway, which induces inflammation. These inflammatory signals, in turn, alter how cells handle energy (Liu Y. et al., 2025). One of the primary benefits of plant metabolites in the treatment of PAH lies in their ability to influence numerous interconnected targets simultaneously.

### 4.5 Epigenetic regulation and the multi-target therapy in PAH

Regarding the pathophysiology of PAH, an increasing number of researchers have found that it is associated with epigenetic dysregulation. This includes abnormal DNA methylation, histone modifications, and altered non-coding RNA expression, which maintain vascular cells in a pathological state (Bontempo et al., 2024; Tai et al., 2024; Ulrich et al., 2024). Plant metabolites with biological activity show promise in modulating these processes. For instance, studies have shown that in PAH, hypermethylation of the BMPR2 gene promoter is among the causes of its downregulation (Bisserier et al., 2021). The lncRNA KMT2E-AS1 has been identified as a driver of EC dysfunction in hypoxia-induced PAH, providing a clear molecular target for intervention with plant metabolites (Bontempo et al., 2024; Tai et al., 2024). Plant metabolites exhibit epigenetic modification-related changes in PAH models, but evidence for direct epigenetic regulation is limited and largely extrapolated from other cardiovascular contexts (Di Giacomo et al., 2023; Rahmani et al., 2024).

**FIGURE 7:**
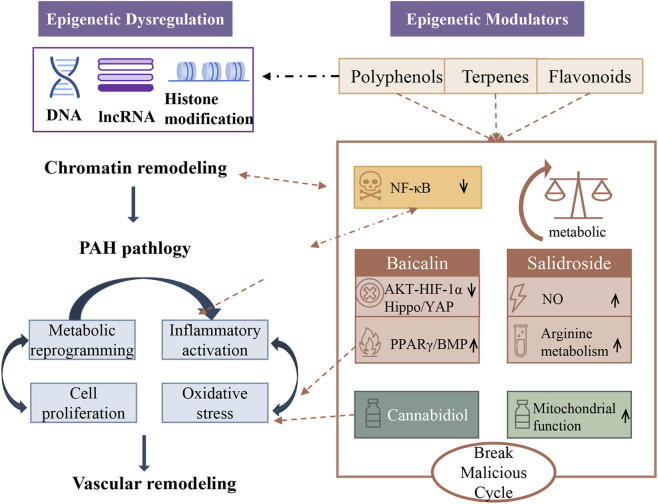
Plant metabolites can simultaneously target interconnected mechanisms such as inflammation, oxidative stress, metabolic changes, and epigenetic functions, demonstrating their potential in the treatment of pulmonary arterial hypertension.

The aforementioned mechanisms have well demonstrated the multitarget properties of plant metabolites. For instance, baicalin exhibits potent efficacy and is capable of simultaneously exerting multiple effects, including alleviating the inflammatory response by inhibiting NF-κB, preventing excessive cell growth by regulating the AKT/HIF-1α and Hippo/YAP pathways, and promoting vascular health by activating PPARγ and BMP signaling (Chen and Wang, 2017; Xue X. et al., 2021; Yan et al., 2019; Zhang et al., 2014; Zhang et al., 2017; Zuo et al., 2021). Salidroside can simultaneously inhibit NF-κB (inflammation), activate BMPR2 (anti-proliferation), restore metabolic balance (AMPK/PPARγ), and induce vasodilation (NO/cGMP) (Li J. et al., 2024). Additionally, cannabidiol effectively alleviates the severity of PAH by enhancing mitochondrial activity, suppressing inflammation, and reducing oxidative stress, indicating its pleiotropic effects (Lu et al., 2021). This holistic, multi-target approach aligns well with the complex, multifactorial pathogenesis of PAH, offering a distinct advantage to single-pathway inhibition (Figure 7).

To conclude, a comprehensive review of the mechanisms underscores the distinct therapeutic benefits of plant metabolites: their capacity to address PAH holistically by concurrently modulating vascular tone, suppressing pathological remodeling, reversing metabolic shifts, and dampening inflammation. Unlike synthetic drugs designed for single targets, this multifaceted action equips them to more effectively disrupt the self-perpetuating pathogenic “vicious cycle” in PAH than drugs with single targets.

Nevertheless, a critical analysis also exposes major deficiencies in current research: most mechanistic claims rely on marker expression changes rather than definitive causal evidence from gene knockdown or rescue experiments; many *in vitro* findings use supraphysiological concentrations that are unlikely to be achieved *in vivo*; and a significant proportion of polyphenolic metabolites are PAINS, which may confound the interpretation of specific pharmacological effects. These limitations highlight the need for more rigorous experimental design and target validation in future studies.

## 5 Discussion

Despite advances in understanding PAH pathogenesis, translating this knowledge into curative therapies remains challenging. The existing treatment of PAH mainly extends the survival time of patients by targeting vasodilation, but it cannot completely solve the fundamental problems, notably vascular remodeling, persistent inflammation, and metabolic disorder (Austin et al., 2024; Chin et al., 2024; Ghofrani et al., 2024; Weatherald et al., 2024). In this context, plant metabolites have emerged as a valuable reservoir for novel drug discovery due to their inherent structural diversity and “pleiotropic” capability to simultaneously modulate multiple pathogenic targets (Li et al., 2022). Although plant metabolites show significant potential for basic research on PAH, numerous challenges remain in translating them from the laboratory to clinical applications. To date, only a few natural products, such as tetramethylpyrazine and sodium tanshinone IIA sulfonate (STS), have achieved limited clinical translation in PAH. However, these examples are exceptions rather than the norm. The vast majority of plant metabolites that are effective in in vitro or *in vivo* studies have not undergone rigorous clinical validation, underscoring the challenge of translating basic research into clinical application.

Our analysis reveals several pervasive methodological issues that undermine the reliability of current claims: (1) Inadequate Model Systems: The almost exclusive use of MCT or acute hypoxia models fails to capture the chronic, inflammatory, and plexogenic nature of human PAH. Positive results in these models have poor predictive value for human efficacy, as evidenced by numerous failed translations. (2) Overreliance on Supraphysiological Concentrations: Many *in vitro* studies on flavonoids and polyphenols employ concentrations (≥10 μM) that are orders of magnitude higher than achievable systemic free concentrations, confusing cytotoxic or non-specific stress responses with therapeutic effects. (3) Descriptive Rather than Mechanistic Studies: The majority of publications report correlations (e.g., ‘compound X downregulates protein Y in a model’) but fail to provide causal evidence through genetic knockout/knockdown, specific pharmacological rescue, or direct binding assays. This is particularly problematic for PAINS compounds, where observed pathway modulation is often a downstream consequence of cellular stress. To facilitate the clinical translation of more natural candidates, several critical limitations in current research must be addressed.

Most efficacy data rely on MCT or hypoxia-induced rodent models (Chen F. et al., 2024; Ding R. et al., 2025; Liu et al., 2024; Lu et al., 2021; Zuo et al., 2021). However, animal models often fail to fully replicate the unique complex plexiform lesions, as well as the heterogeneity and progression of PAH in humans (Yu et al., 2022). This discrepancy may explain why many agents show promise in animals but fail in human trials. Furthermore, most preclinical studies rely on MCT or hypoxia-induced models, which fail to recapitulate key features of human PAH, including plexiform lesions, genetic heterogeneity, and disease progression. This limitation, together with incomplete mechanistic understanding of multi-target interactions, substantially undermines the translational predictability of current findings (Yu et al., 2022). In contrast, the SU5416/hypoxia (SuHx) model more closely resembles human disease and should be preferentially considered in future studies.

A major translational bottleneck for plant metabolites in PAH lies in suboptimal pharmacokinetics and the resulting safety concerns. A typical example is resveratrol, which has strong anti-PAH activity but has an oral bioavailability of less than 10%, a major obstacle to its clinical use (Li et al., 2020; Zhu et al., 2021). Suboptimal pharmacokinetics often necessitate high dosage, thereby increasing the risk of off-target toxicity. Structural modification and the development of novel formulations are crucial to solving this problem. For instance, tanshinone IIA was chemically modified by adding a sulfonic acid group to yield sodium tanshinone IIA sulfonate, which greatly improved its water solubility and enabled it to enter clinical trials (Shang et al., 2012). Similarly, recent advances in nanotechnology, such as DPPC-coated lipid nanoparticles for resveratrol and nanostructured lipid carriers (NLCs) for matrine or aloperine, have shown promise in enhancing lung retention and bioavailability (Li et al., 2020; Liu H. et al., 2025; Xiang et al., 2020). Poor bioavailability often necessitates supratherapeutic dosing, which in turn increases the risk of systemic and organ-specific toxicity, as exemplified by resveratrol-induced renal injury in preclinical models (Shaito et al., 2020). Currently, comprehensive pharmacokinetic analyses and long-term safety assessments are largely missing for most PAH natural candidates. This lack of toxicological data is a major barrier to regulatory approval and clinical trials.

A critical, yet often overlooked, issue in the field is the prevalence of PAINS among studied plant metabolites, particularly polyphenols such as quercetin, resveratrol, and curcumin. As highlighted by Bolz et al. (Bolz et al., 2021) and Magalhães et al. (Magalhães et al., 2021), these compounds can produce false-positive signals in a variety of biochemical and cellular assays through non-specific mechanisms (e.g., redox cycling, protein aggregation, membrane disruption). The uncritical interpretation of *in vitro* data derived from PAINS, especially at high concentrations, has likely led to an overestimation of the specificity and therapeutic potential of many natural compounds. Therefore, mechanistic claims for such metabolites must be interpreted with extreme caution unless supported by dose-response relationships, appropriate counter-screens, orthogonal assays, and, most importantly, evidence of efficacy *in vivo* at pharmacologically relevant exposures. Failure to account for PAINS behavior risks overestimating the therapeutic value of polyphenolic metabolites and may partly explain the poor translation of many promising *in vitro* findings into clinical benefit.

While many studies label plant metabolites as “multi-targeted,” the specific investigations often focus on one or two canonical pathways (Jasemi et al., 2020; Zeng et al., 2023). There is a lack of depth in understanding how a single metabolite or a botanical mixture orchestrates complex networks involving inflammation, mitochondrial dynamics, and epigenetic regulation simultaneously. This comprehensive view is crucial for developing effective treatment methods that address the complex pathogenic factors in PAH. Future studies should move beyond pathway-by-pathway descriptions toward a conceptual synthesis that integrates inflammatory signaling, metabolic reprogramming, and epigenetic regulation into coherent mechanistic frameworks.

Given the multifactorial nature of PAH, combination strategies involving plant metabolites and standard therapies warrant greater systematic investigation. Synergistic approaches may enhance efficacy while allowing for dose reduction, thereby minimizing side effects. However, systematic studies on optimal combinations, dosages, and potential botanical drug interactions are currently scarce and urgently needed.

Most current studies focus on whether plant metabolites play a protective role in the PAH model immediately. Still, little attention has been paid to whether they can reverse established diseases or to their effectiveness and safety over the long term (Silva et al., 2022). Because PAH is a chronic disease that will gradually worsen, we urgently need to find a treatment that can reverse advanced-stage PAH. Advanced drug delivery strategies, particularly lung-targeted and inhalable formulations, will be indispensable for achieving disease-modifying efficacy in chronic PAH. In conclusion, while current single-target vasodilators provide symptomatic relief, they do not cure PAH. Plant metabolites offer a compelling multi-targeted therapeutic alternative capable of addressing vascular remodeling and inflammation. However, the transition from basic research to clinical application is stalled by issues of bioavailability, limited model fidelity, and insufficient safety data. By addressing these gaps through structural optimization, advanced drug delivery, rigorous toxicity testing, and innovative combination strategies, plant metabolites hold the potential to transform the treatment paradigm of PAH.

## 6 Conclusion

Plant metabolites may offer novel multi-target leads for PAH treatment based on preclinical evidence. Still, their translational potential is severely and fundamentally limited by two major factors: (1) the prevalent issue of PAINS-related assay interference, which casts doubt on the mechanistic specificity of many celebrated compounds, and (2) universally poor pharmacokinetic profiles. Further rigorous preclinical research must first employ PAINS-aware orthogonal assays to validate true target engagement before clinical translation can be considered. Research into plant metabolites for the treatment of PAH represents a significant shift in therapeutic strategy. Rather than focusing solely on vasodilation, a more advanced, multi-targeted approach is needed to address this complex, multifactorial disease. PAH is now recognized as a systemic condition involving abnormal vascular remodeling, chronic inflammation, metabolic reprogramming, and epigenetic dysregulation. Traditional single-target therapies demonstrate limited efficacy against this interconnected network of pathophysiological mechanisms. Plant metabolites represent promising hypothesis-generating leads for PAH therapy due to their reported multi-target actions. However, their clinical translation is constrained by PAINS-related assay interference, suboptimal pharmacokinetics, limited target validation, and a scarcity of well-designed clinical trials. Despite these limitations, plant metabolites remain a promising source of novel therapeutic agents for PAH, and addressing the identified challenges will facilitate their successful translation into clinical practice. Future progress will require rigorous orthogonal target engagement assays, exposure-matched *in vivo* studies in relevant models, PK/PD optimization, and carefully designed clinical trials to determine whether these compounds can achieve true disease-modifying effects in PAH.

## Glossary

PAH: pulmonary arterial hypertension
PVR: pulmonary vascular resistance
RV: right ventricle
RVH: right ventricular hypertrophy
SMC: pulmonary artery smooth muscle cell
EC: endothelial cell
PDK1: pyruvate dehydrogenase kinase 1
ECM: extracellular matrix
ET-1: endothelin-1
NO: nitric oxide
PDE5: phosphodiesterase-5
sGC: soluble guanylate cyclase
BMP: bone morphogenetic protein
6MWD: 6-minute walk distance
TCM: Traditional Chinese Medicine
PAINS: pan-assay interference compounds
SASP: senescence-associated secretory phenotype
HIF-1α: hypoxia-inducible factor-1α
BMPR2: bone morphogenetic protein receptor type II
TGF-β: transforming growth factor-β
MAPK: mitogen-activated protein kinase
eNOS: endothelial nitric oxide synthase
PDGF-BB: platelet-derived growth factor-BB
PP2A: protein phosphatase 2A
SeP: selenoprotein P
MCT: monocrotaline
cGMP: cyclic guanosine monophosphate
EndMT: endothelial-to-mesenchymal transition
A2aR: adenosine A2a receptor
PKCα: protein kinase Cα
SOCE: store-operated calcium entry
mPAP: mean pulmonary artery pressure
NETs: neutrophil extracellular traps
STS: tanshinone IIA sulfonate
SIRT1: silent information regulator 1
HMGA2: high mobility group AT-hook 2
TP53: tumor protein p53
[Ca2+]i: intracellular calcium concentration
TRPC: transient receptor potential channels
MLCP: myosin light chain phosphatase
ROCK: Rho-associated kinase
PDGFRβ: platelet-derived growth factor receptor-β
LATS1: large tumor suppressor 1
I-κBα: inhibitor of NF-κB α
TNF-α: tumor necrosis factor-α
IL-1β: interleukin-1β
IL-6: interleukin-6
ROS: reactive oxygen species
NOX1: nicotinamide adenine dinucleotide phosphate oxidase-1
PFKFB3: 6-phosphofructose-2-kinase/fructose-2,6-bisphosphatase 3
AMPK: adenosine monophosphate-activated protein kinase
FAO: fatty acid oxidation
PPARγ: peroxisome proliferator-activated receptor γ
NLCs: nanostructured lipid carriers
EGCG: epigallocatechin gallate
